# An Intelligent Decision Support System for Leukaemia Diagnosis using Microscopic Blood Images

**DOI:** 10.1038/srep14938

**Published:** 2015-10-09

**Authors:** Siew Chin Neoh, Worawut Srisukkham, Li Zhang, Stephen Todryk, Brigit Greystoke, Chee Peng Lim, Mohammed Alamgir Hossain, Nauman Aslam

**Affiliations:** 1 Computational Intelligence Research Group, Department of Computing Science and Digital Technologies, Faculty of Engineering and Environment, University of Northumbria, Newcastle, UK, NE1 8ST; 2 Department of Applied Sciences, Faculty of Health and Life Sciences, University of Northumbria, Newcastle, UK, NE1 8ST; 3 Royal Victoria Infirmary Newcastle upon Tyne, UK, NE1 4LP; 4 Centre for Intelligent Systems Research, Deakin University, Waurn Ponds, VIC 3216, Australia; 5 Anglia Ruskin IT Research Institute, Faculty of Science and Technology, Anglia Ruskin University, Cambridge, CB1 1PF, UK

## Abstract

This research proposes an intelligent decision support system for acute lymphoblastic leukaemia diagnosis from microscopic blood images. A novel clustering algorithm with stimulating discriminant measures (SDM) of both within- and between-cluster scatter variances is proposed to produce robust segmentation of nucleus and cytoplasm of lymphocytes/lymphoblasts. Specifically, the proposed between-cluster evaluation is formulated based on the trade-off of several between-cluster measures of well-known feature extraction methods. The SDM measures are used in conjuction with Genetic Algorithm for clustering nucleus, cytoplasm, and background regions. Subsequently, a total of eighty features consisting of shape, texture, and colour information of the nucleus and cytoplasm sub-images are extracted. A number of classifiers (multi-layer perceptron, Support Vector Machine (SVM) and Dempster-Shafer ensemble) are employed for lymphocyte/lymphoblast classification. Evaluated with the ALL-IDB2 database, the proposed SDM-based clustering overcomes the shortcomings of Fuzzy C-means which focuses purely on within-cluster scatter variance. It also outperforms Linear Discriminant Analysis and Fuzzy Compactness and Separation for nucleus-cytoplasm separation. The overall system achieves superior recognition rates of 96.72% and 96.67% accuracies using bootstrapping and 10-fold cross validation with Dempster-Shafer and SVM, respectively. The results also compare favourably with those reported in the literature, indicating the usefulness of the proposed SDM-based clustering method.

Leukaemia is a type of cancer pertaining to white blood cells (WBCs), whereby abnormal and immature WBCs are produced by the bone marrow and enter the bloodstream. There are two types of acute leukaemia^1^, namely acute lymphoblastic leukaemia (ALL) and acute myeloid leukaemia (AML). Acute leukaemia is usually diagnosed by a morphological analysis of blood slides by haematologists, which is a complex, time-consuming, and costly process^2^. It also requires considerable training and experience. Furthermore, the results often lack of a standardized performance owing to a variety of factors including insufficient expertise or imperfection of the samples^3^,^4^,^5^,^6^. Some digital diagnosis systems were developed to analyse microscopic blood images for leukaemia detection. However, they suffered from a number of limitations, in particular accurate diagnosis of leukaemia requires discrimination of one cell type from another, and of cell nucleus from cell cytoplasm^7^. Indeed, separation of leukaemia cell nucleus with diverse complex irregular morphology from cytoplasm is a challenging task. Research shows that only a few existing clustering algorithms are able to achieve good adaptivity for reliable separation of nucleus and cytoplasm^2^,^8^,^9^. Therefore, the robustness of the existing methods is compromised because of the limitation of the existing clustering algorithms^10^,^11^.

This research aims to address the aforementioned challenges, and to develop an intelligent decision support system for ALL diagnosis using microscopic blood images. It proposes a new clustering algorithm by exploiting the stimulating discriminant measure (SDM) of both within- and between-cluster scatter variances. This clustering algorithm is robust in terms of discriminating cell nucleus from cell cytoplasm of lymphocytes/lymphoblasts with diverse irregular morphology. Figure 1 shows the overall system architecture, which consists of four main stages: (a) WBC identification from blood smear images, (b) nucleus and cytoplasm separation, (c) feature extraction, (d) lymphocyte and lymphoblast recognition.

In this research, marker controlled watershed segmentation is first applied to identify and segment WBCs based on microscopic images. Then, the proposed SDM-based clustering algorithm with discriminant measures is used to perform segmentation of nucleus and cytoplasm. Subsequently, a total of 80 features representing shape, texture, colour, and statistical-based information of the nucleus and cytoplasm sub-images are extracted. A number of classifiers including multi-layer perceptron (MLP), Support Vector Machine (SVM), and ensembles with diverse weighting combination methods are employed to recognize healthy and blast cells. A public ALL image dataset, i.e. ALL-IDB2^12^, is employed in this research for system evaluation. It comprises cropped areas of normal and blast cells obtained from peripheral blood samples of both leukaemia patients and healthy individuals. In comparison with related clustering algorithms and ALL detection systems reported in the literature, the proposed system achieves superior performance in terms of accurate segmentation of nucleus and cytoplasm as well as robust ALL identification.

The contributions of this research include the following:

- In order to perform reliable diagnosis, the system considers both nucleus and cytoplasm in segmentation and feature extraction. This is different from related state-of-the-art applications in the literature which focused purely on nuclei for performing segmentation of WBCs and arriving at the resulting diagnosis^13^,^14^,^15^,^16^.
- The proposed SDM-based clustering takes both within- and between-cluster scatter variances into consideration. This is the main novelty of the proposed system. It overcomes the limitations of the cost function of the classic Fuzzy C-Means (FCM) clustering which focuses purely on the within-cluster scatter variance. It also outperforms other clustering methods including Linear Discriminant Analysis (LDA) and Fuzzy Compactness and Separation (FCS)^17^ for robust identification of cell nucleus and cell cytoplasm.

- A total of 80 features comprising common shape-based descriptors, Gray Level Co-occurrence Matrix (GLCM) textural descriptors, CIELAB colour space, as well as statistical measurements of these descriptors are identified and used to discriminate healthy and unhealthy lymphocytic cells.
- Diverse single and ensemble classifiers are used in the experimental study for lymphocyte and lymphoblast detection. In this research, Dempster-Shafer ensemble achieves the highest accuracy of 96.72% for bootstrap validation whereas SVM with Gaussian Radial Basis Kernel (RBF) achieves an accuracy of 96.67% for 10-fold cross validation.

## Related Work

In this section, we discuss state-of-the-art developments for ALL detection.

### Computerized ALL diagnosis

Since ALL is highly associated with the proliferation of lymphoblast in the bone marrow, accurate identification of lymphocyte and lymphoblast is crucial. Putzu *et al*.^18^ presented an automatic method for WBC classification using microscopic images, where ALL was identified with the support of leucocyte classification. In their research, image cropping and threshold operations were conducted to extract nucleus and cytoplasm into sub-images. Subsequently, 131 features comprising shape, colour, and texture descriptors were extracted from the resulting sub-images. According to Putzu *et al*.^18^, RBF kernel based SVM achieved the best accuracy of 93.2% under 10-fold cross validation for the evaluation of 245 leucocytes from 33 images. In addition, Khasman and Abbas^19^ applied Otsu’s threshold method, median filtering, Canny edge detection and pattern averaging kernel to process 80 lymphocyte images from ALL-IDB2. Extracted image pixels were then used as the inputs for classification using MLP. Three testing strategies with different ratios of training and test sets, i.e., 75%:25%, 50%:50% and 25%:75%, respectively, were employed to evaluate system performance. The 75%:25% training-test strategy produced the highest accuracy rate of 90%, as compared with those from other schemes. Meanwhile, Madhukar *et al*.^14^ proposed a decision support tool for ALL classification, and reported an accuracy rate of 93.5% for SVM using leave-one-out cross validation. In their work, nuclei of the cells were obtained through K-means clustering using the a* and b* components of the CIELAB colour space. Shape-based, texture-based, and Hausdorff Dimension (HD) features of the nuclei were extracted to distinguish normal and blast cells.

### Segmentation of Nucleus and Cytoplasm for Leucocytes

The French-American-British classification systems classify ALL into three subtypes (L1–L3) according to the morphology observation on the nucleus and cytoplasm. Pastel blue and non-granular cytoplasms with closed and clumped nucleus chromatin are usually observed in mature lymphocytes^1^,^20^. For the blasts cells (e.g. different subtypes of ALL), variations in terms of the nucleus to cytoplasm ratio, existence of nucleoli and vacuoles, nucleus and cytoplasm colour, as well as chromatin patterns are observed. Therefore, discrimination of nucleus from cytoplasm and characteristics of nucleus and cytoplasm play significant roles in accurate diagnosis of normal and abnormal lymphocytes. Moreover, according to Rezatofighi and Soltanian-Zadeh^21^, improvement of nucleus-cytoplasm segmentation is the most challenging step that requires the most research efforts.

From the literature, the common techniques adopted for segmentation of nucleus from microscopic images include threshold-based, region-based, edge-based, clustering-based, and morphology-based approaches^22^,^23^. Otsu’s threshold method was employed for nucleus-cytoplasm separation for the recognition of normal and abnormal lymphocytes^4^. Although threshold-based methods are fast in performance, they are not able to perform well with respect to small-variant cluster segmentation. Halim *et al*.^24^ adopted a region growing technique for the retrieval of the nucleus and blast regions, whereby a threshold value of 100 was set to segment the nucleus. Although they reported good results, a similar threshold setting might fail to provide a consistent performance for microscopic images from different databases. Piuri and Scotti^3^ employed an edge detection technique along with morphological operations to detect leucocyte cell membrane. Although image edges can provide rich information for the recognition of image characteristics, edge detection methods tend to be sensitive to image quality and noise^25^. Furthermore, Nemane and Chakkarwar^26^ focused on morphological operations, and employed a watershed algorithm for segmentation of WBCs. Despite that watershed segmentation was able to identify the boundaries with closed and connected regions, over-segmentation could occur^27^.

Besides the above-mentioned techniques, clustering methods recently received much research attention for segmentation of microscopic blood images. Several clustering techniques were investigated by Mohapatra and co-researchers^6^,^9^,^28^,^29^. As an example, Mohapatra *et al*.^2^ employed hard clustering techniques including K-means, K-Medoid, and fuzzy clustering methods such as FCM, Gustavson Kessel and Fuzzy Possibilistic C-means for locating the nucleus in ALL detection. Kernel Induced Rough C-means clustering^8^ and Shadowed C-means^9^ were applied to identify lymphocytes. In addition, Nasir *et al*.^30^ used K-means clustering on the Hue and Saturation components of the Hue-Saturation-Intensity colour space for segmentation of WBCs. Since clustering techniques rely heavily on the principles of intra-class similarity and inter-class separability to perform grouping, the similarity and separability measures play significant roles in determining the resulting cell segmentation quality^31^.

## Clustering, Discriminant Analysis and Limitations

Clustering analysis is widely used to assess the hidden patterns of data samples and organize them into different categories according to the quantitative measurement of distinctiveness^32^. There are two types of clustering: hard and soft clustering. K-means is a popular example of hard clustering that finds the centre of each cluster by minimizing the sum of the square of the distances between sample points in each cluster and their centre, whereby each object in the dataset belongs to exactly one cluster. FCM is a soft clustering algorithm that assigns a membership to each data sample. The key difference here is that a data sample can belong to multiple clusters, and the minimization function employed by FCM is as follows:


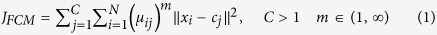


where


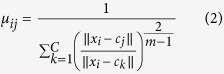



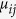
 represents the membership degree of data sample *i* with respect to cluster *j*, whereas *m* is a real value weighting component which is greater or equals to 1. Notice that 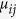 is inversely related to the distance between the data sample and the cluster centre.

Even though the soft partitioning method of FCM through 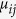 is sometimes more practical for segmenting objects that do not have significant boundaries in an image, FCM is not suitable for non-convex shapes, i.e. noisy data such as very large or very small values that can skew the mean^33^.

Apart from clustering algorithms, data classification techniques such as LDA are generally applied to classify data samples. In LDA, classification is conducted based on two discriminant measures: within-class scatter matrix, 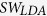 (equation (3)), and between-class scatter matrix, 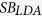 (equation (4)).


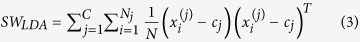



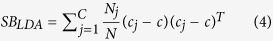


where
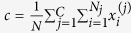
, and *N* is the number of data samples with *N*_*j*_ representing the number of training samples in cluster *j*.

As explained in Li *et al*.^10^ and Theodoridis and Koutroumbas^34^, the criterion of 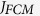
is similar to the trace of the fuzzy within-cluster scatter matrix, which is closely related to the within-cluster scatter matrix of LDA shown in equation (3). As a result, FCM is claimed to consider only the within-class similarity measure^10^. In other words, the exclusion of between-class discriminant measure presents a limitation of conventional FCM. The same issue goes to K-means clustering where the between-cluster criterion is not taken into account in the discriminant measure.

Motivated by the between-class discriminant measure, FCS was proposed by Wu *et al*.^17^ to minimize the within-cluster compactness and maximize the between-cluster separation. Moreover, the objective function of FCS reported in Li *et al*.^10^ is derived as:


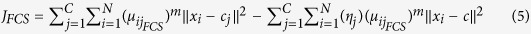


where *j*  = 1, 2, … *C* represents the *j*^*th*^ cluster, and 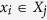 with 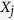 as a set of data samples in the *j*^*th*^ cluster that consists of *N* data samples. Note that 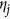 is a weightage parameter as follows:


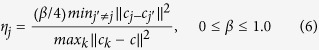


Furthermore, *c*_*j*_ and *c* indicate the centre of cluster *j* and the mutual centre of all clusters, respectively, while 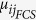 refers to the membership function of FCS^10^, defined as follows:


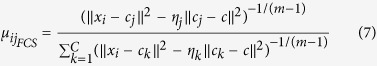


In reference to Wu *et al*.^17^, fuzzy between-cluster scatter matrix, 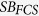, developed on the basis of the fuzzy sample mean, *a*_*j*_, is given as follows^17^:


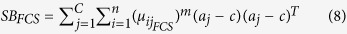


where 
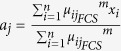
. As such, the proposed objective function of Wu *et al*.^17^, 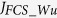, is defined as:


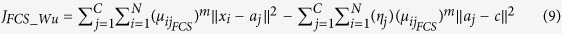


with some slight modifications on 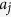, as follows:


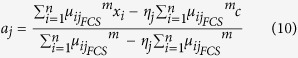


From equation (9), when 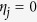, 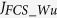 is equivalent to 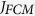. Conversely, when 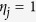, 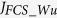 is equivalent to the Fukuyama-Sugeno index.

**Figure 2:**
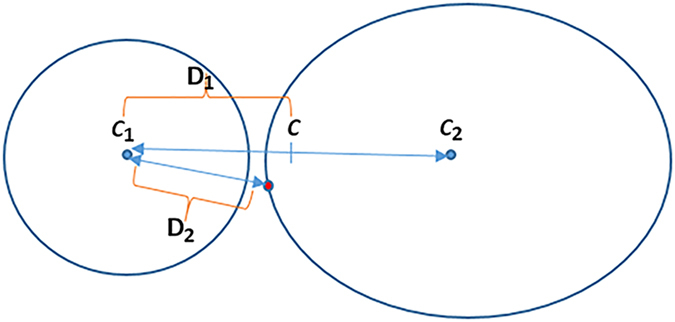
Compact but not well separated clusters (Left: Cluster 1, Right: Cluster 2).

**Figure 3:**
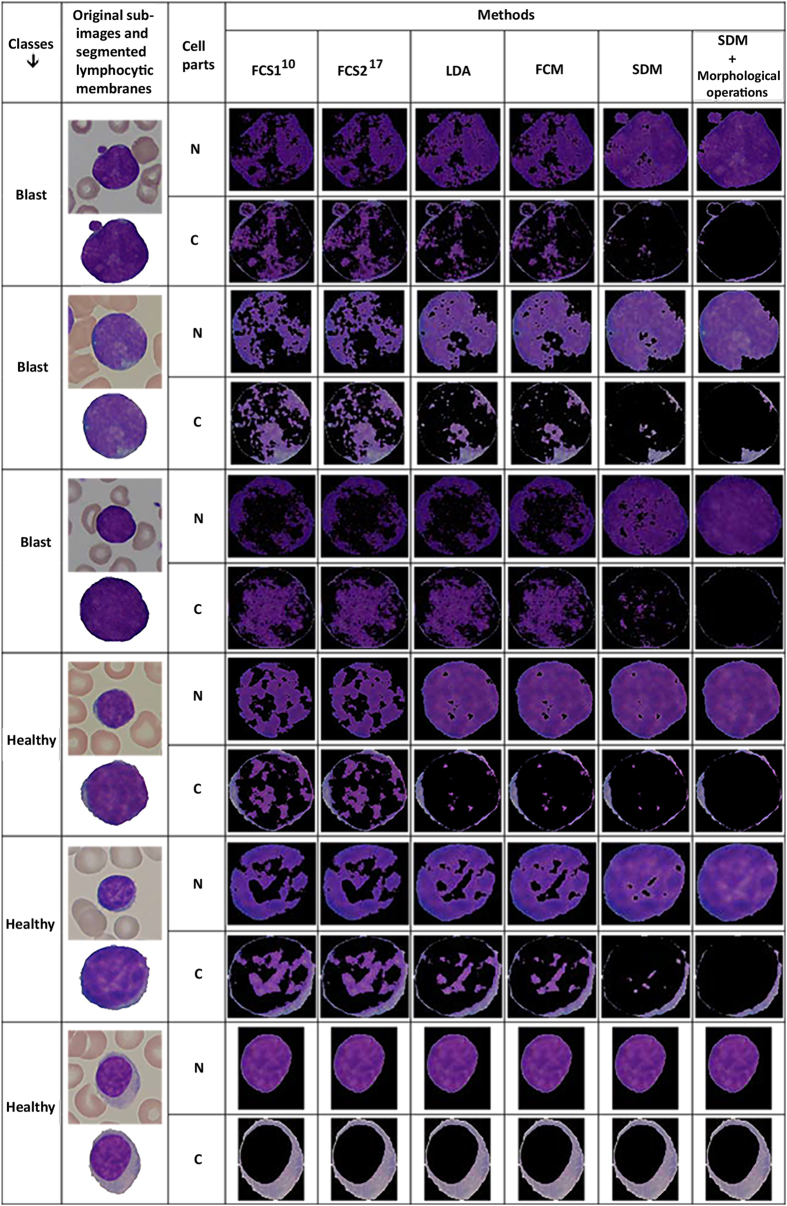
Comparison of the separation of nucleus and cytoplasm between the proposed SDM clustering and other clustering methods.

Although between-cluster variations have been embedded into FCS, it is essential to note that the membership function, 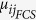 (equation (7)), can be negative when 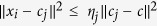. A negative membership value poses an issue to determine the ownership of a data sample in a particular cluster. Wu *et al*.^17^ made a restriction for tackling this issue by proposing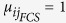, and 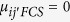, for all 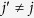, when a negative value is obtained. The assumption is made such that the data sample belongs to cluster *j* completely with 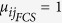, when 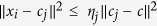. However such an assumption may not be always correct because data samples at the boundary of one cluster can easily be misclassified into another cluster especially when the distribution of data samples along the boundaries of two clusters is close to each other. Figure 2 shows an example of such condition where two clusters are compact but not well separated. In this case, the distance between the red-coloured point of interest and the centre of cluster 1, *c*_*1*,_ (indicated by *D*_*2*_) is smaller than the distance, *D*_*1*_, between *c*_*1*_ and the mutual centre of the two clusters, *c*. According to Wu *et al*.^17^, this data sample should belong to cluster 1. However, the ground truth indicates that it belongs to cluster 2. In this research, such conditions can be observed when the colour and pixel intensity of nucleus and cytoplasm are close to each other, e.g., the first and third blast cells in Fig. 3, whereby the assumption of Wu *et al*.^17^ can mislead the separation of cytoplasm and nucleus. Thus, FCS proposed by Wu *et al*.^17^ sometimes has comparatively less robustness and adaptivity for segmentation of nucleus and cytoplasm with very close cluster scatter measures.

## Method

### Leucocyte Identification

This section focuses on the introduction of the first key stage of this research, i.e. segmentation of WBC membranes from the noisy background of blood smear sub-images with touching red blood cells. This stage, which was reported in our previous research^35^, includes integration of modified marker controlled watershed segmentation and morphological operations for segmentation and identification of WBC membranes. Evaluated with 150 sub-images from the ALL-IDB2 database, it achieved 91.33% accuracy and outperformed traditional marker controlled watershed segmentation. After WBC segmentation, the proposed SDM-based clustering algorithm is applied to each identified WBC, in order to separate nucleus and cytoplasm for disease detection.

### Stimulating Discriminant Measure (SDM)

In this section, a new discriminant measure, i.e. SDM, with both within-cluster and between-cluster assessments is introduced. As observed in equations (1,3 and 5), the within-cluster evaluation from FCM, LDA and FCS is dependent on the summation of 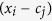 from all data samples in each cluster. Since the centre of each cluster is calculated based on the mean of all data samples in the cluster, those that are not normally distributed skew the value of the within-cluster evaluation. In fact, the data sample with the largest value of 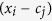 indicates the largest variation from the mean, which indicates that there are no other data samples within the cluster that exceed such a limit. Therefore, in this research, the argument with the maximum value of 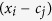, 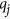, is used to indicate the maximum variation per cluster, and the total within-cluster scatter matrix, 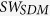, is defined as follows:


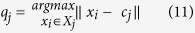



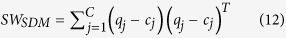


where 
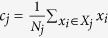
.

**Figure 4:**
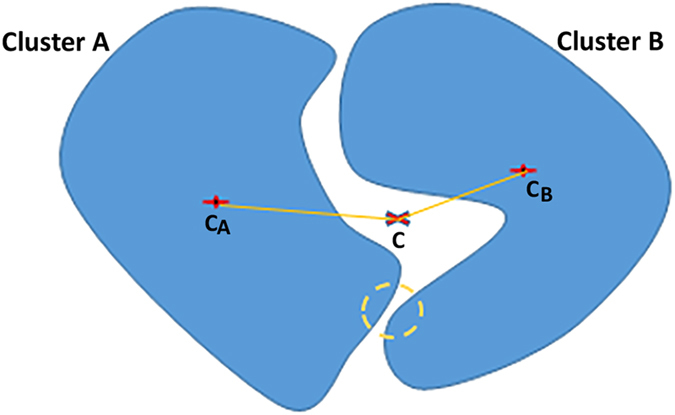
Non-compact clusters.

As for the between-cluster evaluation shown in equations (4) and (8), LDA and FCS take the distance between the centre of a particular cluster and the mutual centre of all clusters, 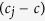, into account. Even though the cluster centres are normally used to give a global view of a specific cluster location with respect to another cluster of interest, the separation between two clusters relies more on the boundary data samples in both clusters. Figure 4 shows the relationship of two non-compact clusters.

In Fig. 4, it is possible that the mutual centre of clusters A and B falls at the location where the two clusters have the larger separation. In this case, distances 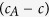 and 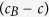 do not provide enough information pertaining to the closest separation between these two clusters, as highlighted in the yellow dashed-line circle. In fact, the closest separation is the most accurate indicator of the separability between the clusters. As a result, the boundary of one cluster that is closer to that of the other cluster reveals more information about the separation between both clusters, A and B. Kuo and Landgrebe^11^ pointed out the importance of using the boundary points for evaluating the scatter matrix in their nonparametric weighted feature extraction (NWFE) method. The between-cluster scatter matrix, 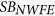, defined by Kuo and Landgrebe^11^ emphasized the cluster boundaries, rather than the mutual centre, for the evaluation of cluster separation based on complicated point-to-point distance weighting assignment, which required the calculation of the distance of each data of cluster *A* to each data of cluster *B*. Such a weighting assignment is computationally heavy when a large number of data samples is involved in both clusters. As an example, if thousands of pixels in a lymphocyte image were to be represented as the data samples during the between-cluster evaluation of hundreds of possible separations of nucleus and cytoplasm, the computational complexity is significantly high.

Motivated by the boundary separation of 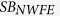 and considering the necessity to reduce the computational complexity, a new between-cluster scatter matrix is defined for SDM in this research. If there are *R* clusters, two clusters out of *R* are evaluated at a time for the separation between clusters. Therefore, the number of possible permutations, *Perm*, from *R* clusters is :


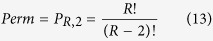


By taking two clusters, *j* and *l*, at a time, let


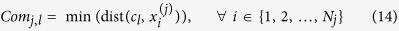


and


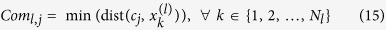


where 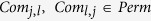, 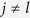, then


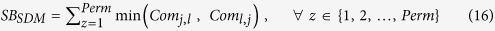


The minimum distance between the data sample in cluster *j* and the centre of the other cluster (e.g. cluster *l*) is used to estimate the nearest point of the respective cluster to the center of the other cluster. In this way, both pairs of minimum distances (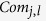 and 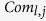) are compared to obtain the closest possible distance between two clusters. Such a process is repeated for *Perm* times depending on the number of cluster combinations. Although the boundary is not uniformly separated, the minimum distance obtained indicates that there are no other segments of the boundary that have a narrower separation based on the estimation towards the center of the other cluster. The proposed 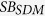 measure avoids tedious point-to-point distance calculation between clusters in 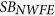 that can exponentially increase the computational complexity during the segmentation. It provides a closer estimation pertaining to the cluster separation than the conventional between-cluster evaluation, which is purely based on the distance of the cluster centre towards the mutual center of all clusters 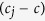, as shown in FCS^17^ and LDA.

### SDM-based Clustering for the Segmentation of Nucleus and Cytoplasm of Lymphocytic Cells

In this research, SDM is embedded into the Genetic Algorithm (GA) to improve the FCM algorithm in separating nucleus and cytoplasm from the lymphocyte/lymphoblast images obtained from ALL-IDB2. Altogether 180 images with 60 normal (lymphocyte) and 120 abnormal (lymphoblast) are segmented in the experiment. The proposed clustering algorithm is performed on the L* component of the CIELAB colour space because the L* component is able to show more differences between nucleus and cytoplasm, whereby nucleus is normally darker owing to the existence of chromatin whereas cytoplasm is relatively brighter. Although the luminence across images varies, the luminence in a particular image during clustering creates a difference between nucleus and cytoplasm.

The proposed SDM-based clustering algorithm aims to improve the segmentation capability of conventional FCM. The algorithm starts with a random initialization of a population, *P*, consisting of chromosomes, 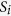, where *i = 1, 2, … k*, that represents the threshold value of three clusters: nucleus, cytoplasm, and the background. During the initialization step, one of the chromosomes, 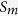, is obtained as a seed from the converged solution of FCM to accelerate the process of optimization, where 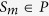. By refering to the threshold value represented by each chromosome, all pixels in the original image are grouped into three clusters, i.e., A, B, and C, which represent clusters of cytoplasm, nucleus, and the background, respectively. In this case, each pixel represents a data sample in a cluster, and a pixel can only belong to one cluster at a time. After separating the pixels, the next step is chromosome evaluation, whereby the chromosome fitness, 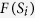, is obtained based on 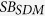 and 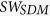, defined as follows:


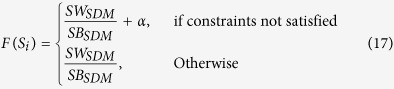


We aim to obtain smaller 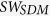 and larger 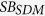, which indicate a higher degree of similarity for within-cluster evaluation and larger separation between clusters, respectively. As mentioned previously, there are cases where the pixel intensity of nucleus and cytoplasm gets very close to each other; therefore implying a greater degree of difficulty to separate both clusters. In this situation, two constraints are used to assist the segmentation process: (i) the nucleus/cytoplasm area should not be less than 10% of the corresponding cytoplasm/nucleus area; (ii) the background area should not be larger than the area of the whole membrane (nucleus + cytoplasm). If the constraints are not satisfied, a penalty value, 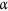, is applied to increase 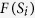.

After evaluating 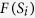, a stochastic universal sampling technique is used to avoid bias during the selection of chromosomes for reproduction. Single-point crossover and mutation are used to produce new offspring with the probability rates of 0.7 and 0.3, respectively. The newly generated offspring are used to divide the pixels into separated clusters (i.e. nucleus, cytoplasm, and background) and further evaluated with the fitness function, 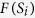. Then, with a generation gap of 0.9, offspring and parent solutions are ranked and merged into the new generation. Based on several trials, the GA is able to converge to a good separation between nucleus and cytoplasm when the maximum number of generations is set to 100. Therefore, the processes of evaluation, crossover, and mutation are repeated until the maximum number of generation (i.e. 100) is achieved.

This research employs SDM as the objective function to guide the search process towards a better segmentation performance. In order to evaluate the discriminant capability of SDM, the segmentation results are compared with those obtained using LDA, FCM, and FCS. For a fair comparison, LDA-based clustering and SDM-based clustering use the same GA settings. The only difference in LDA is that 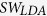 and 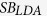 as shown in equations (3) and (4) are used, instead of 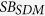 and 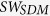. Two types of FCS evaluations are implemented in this research: (i) FCS1 based on Li *et al*.^10^ according to equation (5); (ii) FCS2 based on Wu *et al*.^17^ according to equation (9). In order to avoid negative membership values for FCS as mentioned earlier, equation (7) is modified according to the traditional FCM membership calculation shown in equation (2). This ensures the range of the membership values lies within [0, 1]. The revised membership function of FCS is defined as:


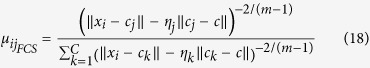


On the subject of 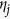, due to the large variation of tuning, the setting of 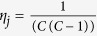 in Yin *et al*.^36^ is adopted, where *C* is the number of clusters^10^.

Overall, this research compares SDM-based clustering with LDA-based clustering, FCM, FCS1, and FCS2 qualitatively and quantitatively. Qualitative comparison is based on visual inspection of the segmented nucleus and cytoplasm, whereas quantitative evaluation is based on a 2-dimensional correlation coefficient between automatic segmentation and ideal segmentation from manual cropping in consultation with haematologists. Equation (19) depicts the formula of the correlation coefficient, *Corr*.


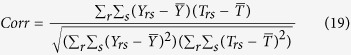


where *r* and *s* refer to the row and column pixels, while 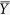 and 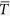 refer to the mean of matrix elements (pixels) in images *Y* and *T*, respectively.

The separation results of nucleus and cytoplasm are discussed in the Evaluation section. Empirical results indicate that SDM-based clustering outperforms other algorithms in terms of nucleus and cytoplasm selection. It is observed that there are high numbers of mis-clustered pixels in the segmented images when the existing clustering algorithms are applied. The proposed SDM-based method, however, only shows very small numbers of mis-clustered pixels (i.e. the so-called “salt and pepper” conditions) in the segmented regions. Such “salt and pepper” conditions can easily be solved by further conducting simple morphological operations focusing on nucleus/cytoplasm to identify small hole areas in nucleus/cytoplasm, fill the holes, and remove the filled pixels from the corresponding cytoplasm/nucleus cluster. Matlab functions “imfill” and “bwareaopen” are used for these morphological operations. The results of SDM-based clustering with and without morphological improvement are compared and discussed in the Evaluation section.

### Feature Extraction

According to Meer *et al*.^37^, the cell size, amount and colour of cytoplasm, shape and chromatin structure are important to characterize lymphocytes. To differentiate normal and abnormal lymphocytic cells, a total of 80 features comprising 16 shape, 54 texture, and 10 colour descriptors are extracted from the segmented nucleus and cytoplasm. The 16 shape descriptors are: cytoplasm area, nucleus area, nucleus to cytoplasm ratio, length to diameter ratio, major axis length, orientation, filled area, perimeter, solidity, eccentricity, minor axis length, convex area, form factor, compactness based on Mohapatra *et al*.^9^, another compactness based on Mohapatra *et al*.^28^, and roundness of the nucleus region. These features mainly aim to extract information with respect to the cell size, nucleus size, nucleus shape, and amount of cytoplasm. As for the 54 texture features, 13 descriptors from the GLCM matrix including correlation, sum of variance, normalized inverse difference moment, sum of average, contrast, difference variance, entropy, cluster prominence, cluster shade, dissimilarity, energy, homogeneity, and normalized inverse difference are computed in four different angles (i.e. 0, 45, 90, and 135). In addition to the GLCM features, skewness and kurtosis are included in the texture descriptors. Chromatin pattern and the existence of nucleoli and vacuole have an effect on the textural information in GLCM. Therefore, these texture descriptors are used to distinguish normal and abnormal lymphocytic cells. Finally, 10 colour features consisting of the mean and standard deviations of the a* and b* components of the CIELAB colour space are evaluated for both nucleus and cytoplasm, along with two descriptors pertaining to the ratio of the mean of a* and b* components between cytoplasm and nucleus.

### ALL Detection and Classification

In this research, we employ a number of classifiers, i.e. MLP, SVM, and ensembles with diverse weighting combination methods, for classifying normal and abnormal lymphocytic cells. Before classification, 80 features comprising texture, colour, and shape-based information mentioned above are scaled into the range of [−1, 1]. These scaled features are then used as the inputs of each classifier for recognizing normal and abnormal lymphocytic cells.

For the MLP, we first conduct a test to find the optimal network topology in order to achieve a good classification rate. Input data normalization is also performed to avoid the dominance of large input values to the learning process. A logarithmic sigmoid transfer function is used as the activation function for the hidden layer, while a linear transfer function is used for the output layer. The Lavenberg-Marquardt algorithm is also used to train the MLP. For the SVM, the RBF kernel is used since it supports nonlinear mapping of data samples and possesses fewer number of hyper-parameters^38^. In order to achieve a good setting of the RBF kernel, the scaling factor, 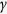, and the soft margin constant, *Co*, are determined using the grid search method^38^. By using exponentially growing sequences, the ranges from 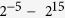 and 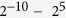 are searched for 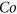 and 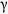, respectively.

In addition to MLP and SVM, ensemble classifiers are implemented with the aim to improve classification accuracy. In this research, a series of ensembles with 9 weighting strategies are employed, i.e., majority voting, minimum and maximum probability, distribution summation, average of probabilities, product of probabilities, Bayesian combination, decision templates, and Dempster-Shafer^39^. To make a feasible comparison study, all these weighting strategies are implemented using the same number of base classifiers with the same setting for each base model. Empirical results indicate that the best accuracy is achieved by Dempster-Shafer, followed by majority voting. Therefore, the results from the Dempster-Shafer ensemble and two single classifiers (i.e. MLP and SVM) are presented and discussed in this research.

Two case studies are conducted in our work: (i) 80 images for comparison with the work of Khashman and Abbas^19^ (ii) 180 images for the overall performance evaluation. The best setting of each classifier for different case studies is given below.

In the first case study, three evaluation schemes comprising different training and test data ratios, i.e. 75%:25%, 50%:50%, and 25%:75%, are used for evaluating a total of 80 images extracted from ALL-IDB2, respectively. The MLP has the following settings, i.e. two hidden layers, each with 8 and 43 nodes for the first and second evaluation schemes; and one hidden layer with 13 nodes for the third evaluation strategy. As for the SVM, the best parameter settings of (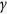
*, Co*) obtained from grid search are (8, 0.5), (8, 4), (16, 32) and for Dempster-Shafer ensemble, there are 10, 11 and 10 MLP base models employed respectively for the first, second and third schemes. Especially, such ensembles are constructed based on the best trade-off between computational complexity and system performance.

In the second case study, two types of validation methods are used: (i) 10-fold cross validation and (ii) 500 bootstrap sampling validation. We employ 10-fold cross validation for evaluating 180 images segmented using the proposed SDM clustering and morphological operations with SVM for ALL classification. In our experiment, 90 images are used for training with the remaining independent 90 images for testing. The settings of SVM are tuned by conducting grid search based on 10-fold cross validation purely on the training set of 90 images. The optimum values of the scaling factor, 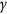, and the soft margin constant, *Co*, are identified, respectively, as 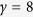
*, Co* *=* *8*. Subsequently, these settings are applied to 90 unseen test images for evaluation.

Although 10-fold cross validation is widely used, over-fitting can occur in some cases since cross validation may over-estimate a classifier’s performance. In order to provide more reliable performance using a more comprehensive evaluation strategy, bootstrap sampling validation is further employed for performance comparison using the MLP, SVM, and Dempster-Shafer ensemble. In this study, we employ .632 bootstrap^40^ with the dataset sampled 500 times with replacement. For each bootstrap sampling, we obtain a training set of 180 images where some images in the original dataset can occur more than once (because of sampling with replacement). The remaining data samples that are not included in the training set form the test set^40^. Finally, the overall accuracy of the bootstrap model, 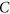, is calculated as follows.


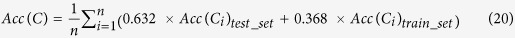


where 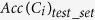 and 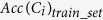 represent the accuracy rates of the model obtained with bootstrap sample *i* when it is tested using test set *i* and the original dataset of 180 images^40^, respectively. In this study, 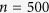 represents 500 times of sampling with replacement.

In order to ensure a similar parameter tuning procedure is used for all the classifiers in bootstrap validation, 10-fold validation tuning as used for the SVM is employed to identify optimal settings of the MLP and Dempster-Shafer. Based on the results, the MLP has two hidden layers with 16 and 30 nodes respectively in the first and second layers. For Dempster-Shafer ensemble, 5 MLP base models are identified. Both MLP and each base model of Dempster-Shafer ensemble share the same topology setting and use a learning rate of 0.1, a momentum rate of 0.8, and a termination error of 0.01, to achieve a balance between accuracy and generalization performance.

## Evaluation

### Evaluation of the Proposed SDM-based Clustering Method

In our experiments, 180 sub-images of 60 lymphocyte (healthy) and 120 lymphoblast (unhealthy) cells extracted from ALL-IDB2 are used for system evaluation. The ground truth of these selected images has been established based on database annotation and further consultation with haematologists from Royal Victoria Infirmary Hospital, Newcastle, UK. Figure 3 shows some examples of the segmented nucleus (N) and cytoplasm (C) samples using different clustering techniques.

The separation results of nucleus and cytoplasm using the proposed SDM clustering method and SDM with morphological operations are the best as compared with those obtained from other prevalent methods. The SDM-based clustering method gives better results in terms of complete separation of nucleus and cytoplasm as well as recognition of the chromatin texture in the segmented nucleus. In particular, the chromatin texture is one of the important features used to differentiate healthy and unhealthy cells. However, when the chromatin texture in the nucleus possesses a similar colour to that of the cytoplasm (e.g. the first and third blast cells in Fig. 3), the extraction of the nucleus becomes very difficult because the chromatin texture tends to be mis-clustered as the cytoplasm by FCS1, FCS2, LDA and FCM. In comparison with these methods, the proposed SDM-based clustering is able to identify most of the chromatin texture in the nucleus with relatively less mis-clustered pixels (“salt and pepper” conditions). To further improve the segmentation from SDM-based clustering, simple morphological operations are conducted on the segmented nucleus and cytoplasm in a vice versa manner to identify small hole areas in nucleus/cytoplasm, fill the holes, and remove the filled pixels from the corresponding cytoplasm/nucleus cluster. As can be observed in the last column of Fig. 3, coupling the SDM-based clustering with the morphological operations manages to produce clean and precise separation results.

In order to validate the separation results of nucleus and cytoplasm in a quantitative manner, a correlation coefficient is used to measure the degree of similarity against manually segmented nucleus and cytoplasm images obtained in consultation with haematologists. The average correlation coefficient of each compared method is shown in Table 1. From the results, the proposed SDM method with morphological operations performs the best with the highest correlation to human segmentation results pertaining to both nucleus and cytoplasm selection. Moreover, SDM achieves better correlation results for both nucleus and cytoplasm, and outperforms other segmentation methods. It is also interesting to note that, although FCM does not include between-cluster scatter evaluation, its robust membership function based on within-cluster scatter is able to produce comparable results to those of LDA, which employs both within and between-cluster matrices but without the implementation of any fuzzy membership. Even though efforts have been made to include between-cluster scatter together with fuzzy membership in the proposal of FCS, the developed fuzzy membership of FCS requires subjective tuning of parameter 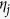. As a result, FCS does not seem to perform well in the experiment when 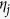 is fixed.

However, FCS2^17^ performs slightly better than FCS1^10^ owing to the consideration of 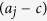, instead of 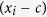 in the between-cluster scatter evaluation, where 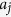 represents the fuzzy sample mean of the *j*^*th*^ cluster while 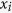 indicates the corresponding data sample and 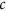 represents the mutual centre of all clusters. The reason is mainly owing to the involvement of all data samples (i.e. all 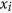) in FCS1^10^ where very large and very small values can affect the evaluation of between-cluster evaluation. The proposed SDM-based clustering does not employ any fuzzy membership, therefore it is not restricted to subjective tuning of parameter 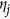. Overall, the proposed SDM method is able to produce more promising segmentation results of nucleus and cytoplasm with a higher correlation coefficient as compared with those from other clustering algorithms.

### Evaluation of ALL Detection

In this research, we employ the MLP, SVM and Dempster-Shafer ensemble for ALL classification. Several evaluation strategies are applied to assess the system efficiency. We compare our research with other related work in the literature. To the best of our knowledge, Khashman and Abbas^19^, Putzu *et al*^18^, and Madhukar *et al*.^14^ have achieved high recognition performances using the same ALL-IDB database. First of all, we analyse the results from our work and those from Khashman and Abbas^19^ because of their impressive system performance. Khashman and Abbas^19^ employed three different schemes of the training and test data ratios for evaluating a total of 80 images extracted from ALL-IDB2, i.e., 75%:25%, 50%:50%, and 25%:75%. In each scheme, a balanced number of normal and abnormal samples in the training and test sets was used. In order to have a fair comparison, we also employ the same three schemes of training and test data ratios to evaluate our system performance using 80 randomly selected images from the ALL-IDB2 database. The detailed comparison results are shown in Table 2. It can be clearly observed that SDM+SVM/MLP/Dempster-Shafer in our study outperforms those of Khashman and Abbas^19^ significantly. The Dempster-Shafer results are better by excel 10%, 18.33%, and 19.9% for the first, second, and third schemes, respectively. Since the MLP is applied in both our work and that of Khashman and Abbas^19^, the MLP results achieved across the three schemes also clearly reveal the strength of the proposed SDM-based method which provides more efficient nucleus-cytoplasm separation to achieve high ALL classification rates.

Putzu *et al*.^18^ and Madhukar *et al*.^14^ are another two related studies in ALL diagnosis. Putzu *et al*.^18^ achieved 93.2% accuracy using SVM with RBF based on 10-fold cross validation, whereas Madhukar *et al*.^14^ achieved 93.5% accuracy with SVM using leave-one-out cross validation. Since SVM was used in both studies, and 10-fold cross validation is a better bias-variance trade-off method as compared with leave-one-out cross validation, we employ 10-fold cross validation for evaluating 180 images segmented using the proposed SDM clustering and morphological operations with SVM for ALL classification. Based on the experimental setting given in ALL Detection and Classification section, we achieve an accuracy rate of 96.67% for 10-fold cross validation using SVM.

Even though 10-fold cross validation is widely implemented, it is undeniable that cross validation might over estimate classifier performance owing to the issue of over-fitting. As a result, a more comprehensive evaluation, i.e. bootstrap sampling validation, is further conducted across MLP, SVM, and Dempster-Shafer in our research.

**Figure 5:**
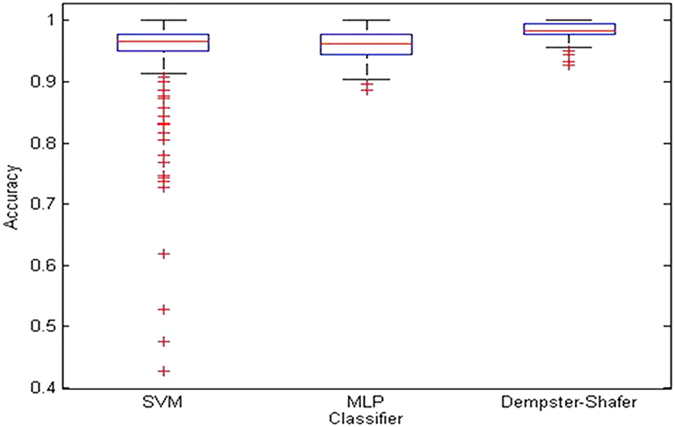
The boxplot evaluation for 500 bootstrap sampling validation.

Table 3 depicts the classifier performances for the original dataset of 180 images for bootstrapping. As can be observed, Dempster-Shafer produces the highest accuracy of 96.72%, followed by MLP and SVM with 95.96% and 95.61% accuracies, respectively. Figure 5 shows the boxplot for 500 bootstrap sampling validation for each classifier. It can be seen that Dempster-Shafer shows a better accuracy distribution with comparatively smaller variations between the 25% and 75% percentiles, as compared with those from the SVM and MLP. Even though there are slight differences in terms of classification rate across different classifiers, significant ALL recognition is observed in both 10-fold cross validation and 500 bootstrap sampling validation. Overall, the proposed SDM clustering segmentation works well, and is able to produce high recognition accuracy for normal and abnormal lymphocytes.

## Conclusion

In this research, we have proposed a decision support system for ALL detection using microscopic images. It integrates a proposed SDM-based clustering method which takes into account both within- and between-cluster scatter variances for robust segmentation of nucleus and cytoplasm. The SDM-based clustering overcomes the limitations of classical FCM which only considers the within-cluster scatter variance. The between-cluster scatter criteria are designed based on the trade-off pertaining to several between-cluster measures (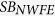 and 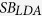) through the application of the GA. The SDM-based clustering method achieves the highest correlation coefficient scores for the selection of nucleus and cytoplasm, and outperforms LDA, FCM, and FCS. A total of 80 feature descriptors are extracted from the segmented nucleus and cytoplasm. These features are used as the inputs to the MLP, SVM and Dempster-Shafer for lymphocyte and lymphoblast identification. For comparison with the work of Khashman and Abbas^19^ using three evaluation schemes, the proposed SDM-based clustering integrated with Dempster-Shafer ensemble achieves the best accuracy rates of 100%, 98.33% and 95%, and outperforms the results in Khashman and Abbas^19^ by 10%, 18.33%, and 19.9% corresponding to the three evaluation schemes. To provide a comprehensive evaluation study on our proposed system, another case study is carried out using 180 images. The results show that 10-fold cross validation together with SVM is able to produce an accuracy rate of 96.67%. In order to prevent over-estimation of the classifier performance, 500 bootstrap sampling validation is further conducted using the SVM, MLP and Dempster-Shafer ensemble. The Dempster-Shafer ensemble achieves the highest accuracy rate of 96.72%. Overall, our system achieves better recognition accuracy in distinguishing normal and blast cells as compared with reported results in the literature.

For future work, since the SDM-based discriminant measure can be used as a fitness/cost function for different optimization algorithms, the SDM-based clustering method with different optimization algorithms will be explored. Ensemble classifiers integrated with clustering techniques will also be explored to detect the arrival of novel unseen classes (e.g. AML) without prior training required^41^,^42^. We also aim to implement the decision support system on a mobile platform to promote instant and robust identification of leukaemia at an early stage.

## Additional Information

**How to cite this article**: Chin Neoh, S. *et al*. An Intelligent Decision Support System for Leukaemia Diagnosis using Microscopic Blood Images. *Sci. Rep*. **5**, 14938; doi: 10.1038/srep14938 (2015).

## Figures and Tables

**Figure 1:**
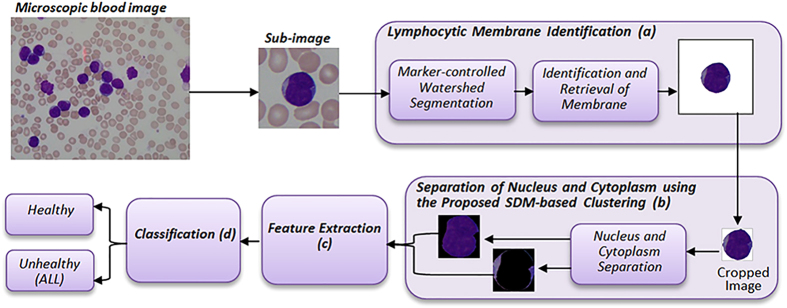
The system architecture.

**Table 1:** The correlation coefficient values of the proposed and several selected clustering methods in comparison to manual separation of nucleus (CorrN) and cytoplasm (CorrC) for 180 sub-images

Methods	CorrN	CorrC
FCS1^10^	0.627	0.624
FCS2^17^	0.633	0.627
LDA	0.773	0.705
FCM	0.774	0.706
SDM	0.841	0.744
SDM + Morphological operation	0.865	0.756

**Table 2:** Comparison of the recognition accuracy according to the three testing strategies used in Khashman and Abbas^19^ (N: Normal, A: Abnormal).

Training & Testing Split	ALL Detection Accuracy			
	Khashman and Abbas^19^ (%)	SDM+SVM (%)	SDM+MLP (%)	SDM+ Dempster-Shafer (%)
Training 75% (30(N):30(A))				
Testing 25% (10(N):10(A))	90	90	95	100
Training 50% (20(N):20(A)) Testing 50% (20(N):20(A))	80	100	96.75	98.33
Training 25% (10(N):10(A)) Testing 75% (30(N):30(A))	75.1	86.67	91	95

**Table 3:** Comparison of ALL detection accuracy using the bootstrap validation method.

Validation Method	Classifiers		
	MLP (%)	SVM (%)	Dempster-Shafer (%)
Bootstrap Validation	95.96	95.61	96.72
